# The social context of severe child malnutrition: a qualitative household case study from a rural area of the Democratic Republic of Congo

**DOI:** 10.1186/s12939-015-0175-x

**Published:** 2015-05-19

**Authors:** Hallgeir Kismul, Anne Hatløy, Peter Andersen, Mala Mapatano, Jan Van den Broeck, Karen Marie Moland

**Affiliations:** Centre for International Health, University of Bergen, 5009 Bergen, Norway; Fafo, Box 2947, Tøyen, 0608 Oslo, Norway; Department of Geography, University of Bergen, 5020 Bergen, Norway; School of Public Health, University of Kinshasa, Kinshasa 1, Democratic Republic of Congo

**Keywords:** Malnutrition, Marasmus, Kwashiorkor, Food security, Subsistence agriculture, Social inequality, Social capital, The Democratic Republic of Congo

## Abstract

**Introduction:**

The magnitude of child malnutrition including severe child malnutrition is especially high in the rural areas of the Democratic Republic of Congo (the DRC). The aim of this qualitative study is to describe the social context of malnutrition in a rural part of the DRC and explore how some households succeed in ensuring that their children are well-nourished while others do not.

**Methodology:**

This study is based on participant observation, key informant interviews, group discussions and in-depth interviews with four households with malnourished children and four with well-nourished children. We apply social field theory to link individual child nutritional outcomes to processes at local level and to the wider socio-economic environment.

**Findings:**

We identified four social fields that have implications for food security and child nutritional outcomes: 1) household size and composition which determined vulnerability to child malnutrition, 2) inter-household cooperation in the form of ‘*gbisa* work party’ which buffered scarcity of labour in peak seasons and facilitated capital accumulation, 3) the village associated with usufruct rights to land, and 4) the local NGO providing access to agricultural support, clean drinking water and health care.

**Conclusions:**

Households that participated in inter-household cooperation were able to improve food and nutrition security. Children living in households with high pressure on productive members were at danger of food insecurity and malnutrition. Nutrition interventions need to involve local institutions for inter-household cooperation and address the problem of social inequalities in service provision. They should have special focus on households with few resources in the form of land, labour and capital.

## Introduction

Malnutrition contributes significantly to mortality in children under five years and in 2011 it was estimated that about 45 % of child deaths could be attributed to malnutrition [1]. Marasmus and kwashiorkor are both forms of severe malnutrition and have especially high mortality rates [2, 3]. While Marasmus is characterised by extreme wasting, Kwashiorkor is characterised by oedema and the aetiology of this disease is still uncertain. Child malnutrition in the form of stunting, wasting, underweight and severe malnutrition has significant implications for healthy human development in terms of motor skills, and cognitive and social development [4–7]. There are several pathways to malnutrition. Poor diet and illness have been identified as immediate factors that contribute to the development of malnutrition, food insecurity has been identified as an intermediate factor, and socio-economic conditions as underlying causes [8]. Growing social inequalities and determinants of health attracted special attention during the last decades [9, 10]. In low and middle-income countries there is evidence of increasing social inequalities in child nutrition with the highest rates of malnutrition being found in the poorest segments of the population [11–14]. Urban–rural inequalities in child malnutrition are frequently found with a higher risk among the rural population [15–20]. The factors that affect nutrition in rural and urban areas differ and a higher reliance on agriculture and natural resources, and a lesser dependency on cash income are characteristic of rural areas. [15]. The majority of rural people in low-income countries live on small farms of less than one hectare and agriculture is the foremost provider of food and the principle source of income [21–23]. Sub-Saharan Africa is more dependent on agriculture than any other region in the world and small-scale agriculture is particularly important [24]. In areas that strongly depend on agriculture there is a close linkage between agriculture and nutrition. Agriculture as a source of food is the most direct pathway between agriculture and nutrition [25]. The urban–rural gap in malnutrition has also been attributed to factors such as education, access to quality food and availability of health services [15, 17, 18, 20]. Maternal education, especially education at secondary level, is considered to be among the most important factors that explain urban rural differences in malnutrition [17, 18, 20]. Besides investigating inter-household inequalities, several studies have examined intra-household inequalities in nutrition. While studies from South Asia have reported discrimination against girls in food allocation and malnutrition being more common among girls than boys [26–29], research from sub-Saharan Africa on gender inequalities in nutrition is inconclusive [30–33].

The Democratic Republic of Congo (the DRC) is among the countries in the world with the highest rates of child malnutrition [1, 34]. Although malnutrition is widespread in all provinces there are important geographic variations and the occurrence is significantly higher in rural than in urban areas [34, 35]. While the prevalence of stunting in rural areas in 2013 was 47 % it was 33 % in urban areas. In the rural areas of the DRC subsistence agriculture is the major livelihood for the majority of the households [36, 37]. Currently there are several constraints to subsistence production: farmers cultivate small land-holdings, they rely on traditional cultivation technologies, have limited access to agricultural input, infrastructure is poor and pressure on the productive population is high [35, 36, 38]. In the context of civil war the subsistence agricultural sector has also been seriously neglected by the government and development agencies [37].

In small-scale agricultural communities the household is typically the unit responsible for food production and consumption [39, 40]. Hence, the social organisation of the household has important implications for food and nutritional security [39]. In this paper we explore how household characteristics, access to land and inter-household cooperation affect food security and vulnerability to child malnutrition in an environment where subsistence agriculture is dominant. Using the Bwamanda area, located in a rural part of western DRC as a case, we aim to describe the social context of food production and nutrition, and explore how some households succeed to ensure that their children are well-nourished while others do not.

## Methods

### Study setting

#### The Democratic Republic of Congo (the DRC)

**Fig. 1:**
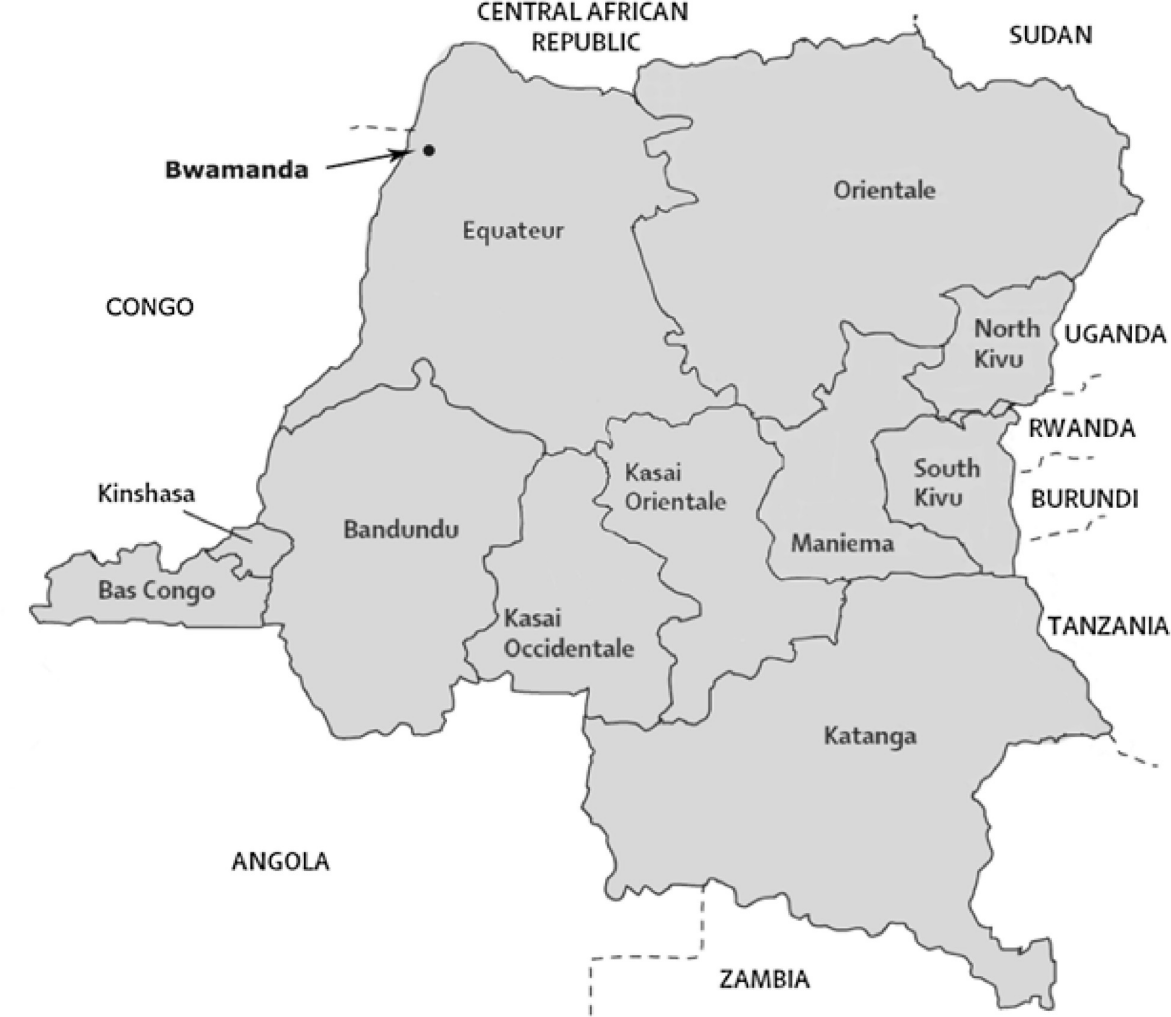
Map The Democratic Republic of Congo, provinces and location of Bwamanda

The DRC is located in south-west central Africa and is the second largest country in Africa. It is divided into ten provinces and one city province (see Fig. 1 map). In terms of natural resources it is among the richest countries in the world and has a diversity of mineral and forest resources [41]. It also has an environment that is favourable for agricultural activities and allows for two harvests per year [42]. Despite the DRCs wealth in natural resources, its population is among the poorest in the world and because of its poor scores with regards to income, health and education it is ranked as second to last according to the Human Development Index [43]. There is a rural–urban gap in poverty disfavouring rural areas where eight out of ten households are living below the poverty line of 1.25 dollars a day while in urban areas it is less than seven out of ten [41]. Since 1997 and until now the political situation in the country has been characterised by civil wars and corruption. The death toll of the civil war, 1998 – 2004, has been estimated to 3.9 million [44]. The conflicts have restricted the country’s ability to promote development and it is still strongly dependent on foreign aid [37, 45].

#### The Equatorial province

The Equatorial Province where this study was undertaken is situated in the north-west part of the country. The province covers an area of 403.292 km^2^, 17 % of the DRC, and is composed of five districts. It has a population of five million. According to a UNDP report from 2009 as much as 94 % of the population was living below the poverty line of 1.25 dollars a day, the province was the poorest in the country [46]. The proportion of children suffering from malnutrition in 2013 was high: 57 % of the children under five years of age were stunted and 7.6 % wasted [34]. With prevalence in 2007 of 10.5 %, this province had the highest proportion of children with kwashiorkor [47].

#### Bwamanda area

The study was carried out in the Bwamanda area in the north-west of the Equatorial Province. Bwamanda village and its surrounding villages form the Bwamanda area, with a total population of about 209,000. The Ngbaka is the dominant ethnic group. Their principle livelihood is subsistence agriculture [48–50]. Bwamanda is a large village that has grown into a centre with a marketplace, a hospital and associated health centres. The Bwamanda hospital operates as a first referral hospital for the health district/zone of Bwamanda. Currently, the local NGO, Centre de Développement Intégral Bwamanda (CDI-Bwamanda) is responsible for providing social services in the area.

### Data collection

This qualitative study is part of a larger project on malnutrition in the Bwamanda area [49, 51–53]. The data were gathered during three fieldwork visits: October and November 2012- February- March 2013, and in November 2013. Data collection and translation was done with the assistance of a secondary school teacher teaching English and French. Prior to data collection the first author provided him with a three days training in conducting semi-structured interviewing and organising group discussions. We used purposive sampling of households of two groups based on the criteria of (1) recent history of severe malnutrition and hospitalisation of a child in the household, and (2) absence of a recent history of malnutrition among children in the household.

We met with nurses and physicians working in the hospital and identified four cases of children under-six-years who had been hospitalised for, and later recovered from marasmus and kwashiorkor. With the assistance from nurses at health centres, four households with children under-six-year who had not suffered from malnutrition were also selected. During vaccination the health centres had conducted anthropometric assessment and the results had been registered on the children’s health cards. The nurses used this information to identify children that had been assessed using normal weight for age charts and found not to be underweight. We had thereby identified eight households in five different villages which were eligible for inclusion in the study. These eight households comprised 12 girls and 17 boys below six years, 24 girls and 11 boys above six years and 24 adult women and 21 men.

Triangulation of data collection methods were applied (see Table 1). Participant observation was used both to map agricultural activities, and the spatial organisation of the villages, and within households to understand household composition, organisation of food production consumption. Semi-structured interviews were conducted with the fathers and mothers of the children as well as other adult household members of all selected households. During the interviews, social factors associated with food production and the children’s nutritional status were discussed. To obtain information about socio-economic conditions and social service provision, key informant interviews were conducted with local leaders including the village chief, village secretary, chief assistants and older respected women.

**Table 1:** Number of informants, methods used and dates for interviews and discussions

Informants	Number of informants	Methods	Dates
Informants from households with malnourished children	10	In-depth interviews	February/March 2013
		Participant observation	November 2013
Informants from households with well- nourished children	10	In-depth interviews	February/March 2013
		Participant observation	November 2013
Village leaders	5	Two focus group discussions	February/March 2013
		In-depth interviews	November 2013
Bwamanda hospital and health centre staff	4	In-depth interviews	February/March 2013
			November 2013
Primary and secondary school teachers	3	In depth-interviews	November 2013
CDI Bwamanda representatives	4	In-depth interviews	November/February 2012
			February/March 2013
			November 2013
Total number of informants	36		

Finally, two focus group discussions were held with male and female leaders to gain a better understanding of the Ngbaka socio-economic organisation including social differentiation. The observation, interviews and focus group discussions were all carried out in the Ngbaka language and translated by the interpreter. All interviews were tape recorded. After each interview the interpreter and the first author went carefully through the tape-recorded interview. The interpreter translated each point raised in the interview orally into English and the first author took notes. The meaning and interpretation of the interview data were then extensively discussed. Field notes from observation and informal conversations were kept in addition to reflection notes from each day of the fieldwork. These served as guides for analysis.

### Field theory and data analysis

#### Field theory

A social field is a domain of social life that has its own rules of organisation and unique characteristics that generate the conditions for the individuals who live in a society [54]. The social fields can be identified in terms of extension in social space, time, number of people and its distinctive characteristics [55, 56]. In the process of identifying social fields the spatial aspect of social fields is in particular important [54, 57]. The fields are interconnected and the theory enables an analysis of how events at the local level are connected to processes at the macro level [56]. The concept of the social field can be used to study the relationship between social factors on different levels that shape food production, consumption and nutritional outcomes. Concretely the theory allowed us to examine how the household as a micro level domain is linked to other social domains and how the dynamics between these domains produce social inequalities in nutritional outcomes. In our field analysis we put emphasise on social organisational aspects of social fields and do not analyse fields as socio-cultural entities with their own forms of communication. We therefore do not analyse meaning and our approach differ from a qualitative content analysis.

### Data analysis

During the field work we observed and discussed with key informants how location related to food production and consumption. We examined this relation with respect to smaller areas including the compound, the neighbourhood, the village and larger areas such as agricultural fields, natural areas in the vicinity of the villages and the Bwamanda area. In this manner with could identify separate bounded areas that we term social space. We identified major characteristics of social activities by describing the context of production and consumption. Making linkages between social space and activities with their own characteristics we could begin to delineate separate fields. Through the description of the household cases we further singled out field characteristics and fields’ implications for nutrition. By having identified the social fields we were able to present factors that could be easily compared and analysed. We performed cross case-case comparisons and analysed how the fields had different implications for food security and nutritional status. Household cases were used to show the linkage between social organisation and nutrition and we therefore did not use quotes to highlight this relation.

### Ethical issues

Ethical clearance was provided by the Regional Committee for Medical and Health Research Ethics, Western Norway and by the Ethical Committee at the School of Public Health, University of Kinshasa, the Democratic Republic of Congo. For ethical reasons we recruited children who previously had suffered from malnutrition. In regards to the fieldwork and data collection, an information sheet and informed consent form were prepared in the Ngbaka language. We explained the content of the form to each participant and obtained informed consent before starting any data collection including consent to record the interviews. Finally, although the households are described in detail in the findings section, we strived to keep names and location confidential.

## Findings

The first section gives an account of the Ngbaka socio-economic organisation and we describe characteristic activities relating to food production, consumption and nutrition. The description of socio-economic context is used as a backdrop for organising the household cases and links between the social context and nutritional outcomes. In the second section eight household cases are presented: the first four are households with a history of severely malnourished children and the last four are those with well-nourished children. Table 2 gives an overview of household cases structured in accordance with the description of the socio-economic context.

**Table 2:** Overview of social fields with implications for household food security and child nutrition

Case no.	Children’s nutritional status	Social fields			
		Household	Gbisa	Village	Local NGO
		Households with malnourished children			
1	Marasmus	Nuclear family	Used male *gbisa* for land clearing	Rights to plots for maize and cassava cultivation.	Marasmic child treated at the hospital
			No use of female *gbisa*	Land redistributed by village chief	No access to safe water
2	Marasmus	Nuclear family	No use of *gbisa*	No agricultural land	Marasmic child treated at the hospital
					No access to safe water
3	Kwashiorkor	Medium sized – extended household	No use of *gbisa*	Rights to agricultural land for maize, cassava and groundnut cultivation	Kwashiorkor child treated at the hospital
					No access to safe water
4	Kwashiorkor	Large extended household – three generations	No use of gbisa	Rights to several plots for maize, cassava and groundnut cultivation	Kwashiorkor child treated at the hospital
					No access to safe water
5	Well-nourished	Medium sized monogamous household	Use of male and female *gbisa* for agricultural activities	Usufruct rights to plots for maize, cassava and groundnut cultivation	Use of health centre services including counselling for infants and toddlers
					Access to safe water provided by CDI-Bwamanda
6	Well-nourished	Large polygamous household – three generations	Use of male and female *gbisa* for agricultural activities Use of “groundnut” gbisa for capital accumulation investment in bicycle	Usufruct rights to several plots for maize, cassava, groundnut and palm oil cultivation	Use of health centre services including counselling for infants and toddlers
					Access to safe water provided by CDI-Bwamanda
7	Well-nourished	Large polygamous household	Use of *gbisa* for capital accumulation – investment in cattle	Usufruct rights to several plots for maize, cassava, palm oil cultivation and growing fruits	Use of health centre services including counselling for infants and toddlers
					Benefit from project combatting sleeping sickness
					Benefitted from hygiene project
					No access to safe water supply, but involved in planning drilling of new deep water well to be provided by CDI-Bwamanda
8	Well-nourished	Large polygamous household	No use of *gbisa*	Usufruct rights to several plots for maize, cassava, beans and palm oil cultivation	Use of health centre services including counselling for infants and toddlers
			Employ cash labourers and school children as an alternative to *gbisa*		
					Took advantage of CDI-Bwamanda facilitating transport and sale of maize to Kinshasa
					Benefitted from project combatting sleeping sickness
					Benefitted from hygiene project
					Access to safe water supply provided by CDI-Bwamanda

### Ngbaka socio-economic organisation

#### Village leadership and access to land

The Ngbaka live in villages whose names typically begins with the pre-fix *bo* which means descendant, followed by the name of the founder of the village. Each village has a chief (*capita*) who is supported by several assistants. Land administration is a major task of the village leadership with the leaders negotiating in land conflicts and being responsible for land redistribution. In Bwamanda, land is under a traditional community-based property system and individual farmers are entitled to usufruct rights. In accordance with the Ngbaka patrilineal descent system land rights are transferred from father to son. In order to uphold usufruct to land the family is required to continuously cultivate it and reside in the village.

#### Food production

The Ngbaka farmers produce their staple foods through shifting cultivation and a household’s planted land commonly covers less than one hectare. Maize and cassava are staples and groundnuts and palm oil are major cash crops. Some farmers also grow crops such as taro, sweet potatoes, pigeon peas, beans and various vegetables and fruits. Farming techniques are very traditional; all operations are done by hand, farmers do not have access to draught animals and fertilisers are unavailable. Agricultural fields are cleared during the first two months of the year, planted and weeded in April up to the beginning of June. The first harvest of maize takes place in June and the other in November, while farmers begin to harvest cassava in October. After three to four years the soil is exhausted and land is left fallow for several years. Fallow land is sometimes used for oil palms and tree crops. To supplement crop production poorer farmers keep poultry and guinea pigs while better-off farmers raise pigs, sheep, goats and cattle.

In addition to agriculture the Ngbaka hunt, gather wild food and fish. Men hunt whereas women gather wild food, but both men and women fish. While men fish using rods, nets and traps, women catch fish in temporarily dammed pools as they drain out. Natural resources in Bwamanda are widely dispersed; agricultural fields can be located up to 4 h walk from the homesteads and in the dry season people go on foot for several hours to fish in the rivers.

#### Food consumption

The Ngbaka normally eat two meals per day with a main meal that typically consists of *ka,* a stiff porridge made from cassava and maize flour. Porridge is served with a stew of cassava leaves, sometimes enhanced with fish and groundnuts. In between meals, adults and children drink tea with sugar and eat various fruits. Infants are predominantly breastfed up to three months old, at which point solid food is introduced to complement breast milk. Breastfeeding normally continues for up to three years. Early complementary food consists of gruel typically made from *ka* and cassava-leaf stew. During meals household members are served the same food, but split into groups; women and young children in one group, older children in another and men in the third.

#### Labour organisation and inter-household cooperation

Agricultural work is carried out by the household members and has a gender-based division of labour. Men are responsible for clearing land and women do most of the work during weeding and harvesting. Farmers also mobilise labour and capital through the traditional *gbisa*. These are reciprocal groups consisting of close kin and neighbours that are mobilised to solve tasks that the household unit have difficulties solving alone such as land clearing and timely weeding. During *gbisa* the host serves *ka* and cassava leaves and farmers who can afford it serve meat, fish and palm wine. Farmers underline the importance of gbisa and, by organising such groups, they are able to achieve a good harvest and provide household members with sufficient food. Male *gbisa* is also organised for capital accumulation with groundnut *gbisa* being the most common example. In the first year, the person who initiated the group receives an agreed upon number of sacks of groundnuts from group members, and in the following years others obtain sacks of groundnuts on a consecutive basis. Capital from groundnut *gbisa* is typically invested in livestock, bicycles and sewing machines. There is also a second form of *gbisa* for capital accumulation whereby the group establish a revolving fund that provides cash in a sequential manner to its members.

#### Household organisation

Our study illustrates how households vary in size and composition. There are large multi-generation households and households that are large partly as a result of influx of children from households that have ceased to exist. Other households are large due to polygamy. Small households comprise nuclear families where the sons have broken away from their family and established their own households. The Ngbaka are patrilineal and practice patrilocality, with the wife moving to her husband’s father’s household after marriage. Local people use wealth to differentiate between households and distinguish three categories—relatively wealthy, average and poor—using the following terms in Ngbaka. The relatively wealthy cultivate a variety of cash crops including palm oil and many have become wealthy through *gbisa*. The averagely wealthy are able to produce enough food for their household members during normal years, while the poor are not. The poor are also characterised by their limited capacity to participate in *gbisa* as a result of not being capable to provide the food required to host a gbisa and being considered by other farmers as unable.

#### Service provision

In Bwamanda the NGO, CDI-Bwamanda, has filled the gap in public service provision. Services provided by the NGO include health care, access to safe drinking water and agricultural support. Currently the organisation runs the Bwamanda hospital and associated health centres. In order to improve access to drinking water the NGO has developed a number of deep borehole wells. CDI-Bwamanda has made several efforts to stimulate agricultural growth and provided farmers with improved planting material, facilitated transport of maize for sale in Kinshasa and promoted coffee as a cash crop. A tsetse control program has permitted cattle raising, which was difficult earlier due to trypanosomiasis (sleeping sickness). Due to a decline in financial assistance from international donors over recent years, the organisation has had to scale down its operation and now concentrates on health services. In spite of this CDI-Bwamanda health services are inadequate because of a shortage of qualified staff, basic equipment and essential medicine including ready-to-use therapeutic food to treat child malnutrition. A few years back the hospital received funding for developing local therapeutic food, but funding for this project has ceased.

### Case studies

It is in the context of the Ngbaka socio-economic organisation that the household cases must be understood and we have structured the case narratives so that the relationship between the social environment and nutrition becomes more evident. For each case we have therefore described food production, household organisation, inter-household cooperation and household access to social services.

### Households with children with a history of marasmus

#### Case 1

A three-year-old boy was brought to the health centre by his parents in December 2012. He was referred to the hospital, where he was diagnosed with marasmus. Before the child was hospitalised for marasmus the household had insufficient food, and all it could provide the child with was *ka* and cassava leaves. The household comprised five members including the father (29) the mother (28), the boy (3) and twins, 17 months. Another son had died from marasmus a few years back, aged three. The parents of the child first lived with the father’s family, but as the household grew larger, they decided to move and find their own place. Following land redistribution carried out by the village chief, the couple obtained access to a homestead and agricultural land, with one plot for maize and one for cassava. For clearing the land the father involved a *gbisa*. With their children being so sick, the parents had not been able to spend the necessary time tending to their two plots, and consequently weeds suppressed their fields resulting in poor harvests. Caring for the sick boy and breastfeeding the twins had made it hard for the mother to find time for fishing. Buying fishing hooks was also an unaffordable expense and the father could therefore not go fishing. They failed to produce a sufficient amount of food and had no stores of maize and the family had to subsist on cassava from the fields. Their opportunity to supplement their cassava- and maize-based diet with fish was severely curtailed. Facing acute food scarcity the family had to rely on food provided by relatives living nearby. They also lived on the outskirts of the village and had no access to safe drinking water provided by CDI-Bwamanda.

#### Case 2

A boy aged 16 months was brought to the health centre in January 2013, where he exhibited signs of severe malnutrition. The health centre referred the boy to the hospital in Bwamanda, where he was diagnosed with marasmus. The family spent one whole day walking to the hospital. In order to pay for the hospital expenses, the boy’s mother pledged the only saucepan in the household. Although the boy had not completely recovered he was discharged from the hospital. The health centre in the village continued to provide care for the child until he gradually recuperated. Before the child was hospitalised for marasmus the household had insufficient food, and all it could provide the child with was *ka* and cassava leaves. The household comprised three members; the mother (17), the father (25) and the malnourished boy. In 2011 the family lived in the father’s village. They had moved to this village in order to seek patrilineal rights to land. Many years ago the boy’s parental grandfather had left this village in order to marry a woman from a village outside the Bwamanda area. Moving back to his village of origin, the father had acquired an agricultural plot from a relative. After the land had been cleared, the relative demanded it back. Without any land, the father started harvesting oil palm fruits on the fallow land of other farmers. He thus acquired a very small income from selling palm oil. His wife also received cassava root and leaves as payment for working as a labourer on another farmer’s field. Since the household had no access to land they did not participate in *gbisa.* The household had no stores of grain and they had no relatives who would help them with food. In addition they had no access to safe water provided by CDI-Bwamanda.

### Households with children with a history of kwashiorkor

#### Case 3

A three-year-old boy was brought to the health centre by his parents and the centre referred the boy to the hospital, where he was diagnosed with kwashiorkor. In the period before the boy fell ill from kwashiorkor he had been eating mostly *ka* and cassava leaves. The household was composed of 12 members including the father of the boy (42), his second wife (32), the father’s mother, five adolescent girls and four preschool children. Two of the preschool children, including the boy who had suffered from kwashiorkor, were children of the first wife of the father. The first wife had left and given the father the responsibility of taking care of the two children. The household cultivated two plots on which they grew maize and cassava for subsistence, and groundnuts as a cash crop. Because the fertility of the land in use was rapidly declining, the father wanted to clear more fallow land. With only one adult male member, there was inadequate labour within the household to clear additional land. Income from the groundnut sale was spent mostly on school fees for the older children and there was no surplus for hiring labour. Involving the *gbisa* in clearing the land was also said to be impossible because it required the household to provide fish for feeding the group members during the workday. The father said he did not have enough cash to buy fishhooks and could not afford to purchase fish. He and his second wife reported that because they were unable to clear more land they were incapable of providing a more diverse diet for their household members. The household had no access to safe drinking water provided by CDI-Bwamanda, and they fetched water from a reservoir that was also used for washing clothes.

#### Case 4

An 18-month-old girl was brought to the health centre by her parents in January 2013. She was referred to the hospital where she was diagnosed with kwashiorkor. During the period before she was hospitalised she had suffered from diarrhoea, vomiting and fever. The girl was breastfed complemented with gruel that contained fish. The parents explained that the girl became malnourished because she drank contaminated water from a hand-dug well. The household comprised 30 members from three generations, among them the father of the girl (34), the mother (34), and seven children. They cultivated several plots of land and in addition to producing staple crops for subsistence they obtained cash income from selling maize and groundnuts. They were dependent on household labour, but occasionally hired labour for clearing fallow land. They had also invested in 12 goats used for meat. In addition to *ka* and cassava leaves, they had fish almost every day, as well as chicken and other meat a couple of days per week. They had no access to safe drinking water, from CDI-Bwamanda and they collected water from a hand-dug well that was deemed unsafe and several household members had become sick after drinking water from this source.

### Households with well-nourished children

#### Case 5

This was a monogamous household consisting of 15 members in total. The household head lived with his wife and his sister, two adolescents and ten younger children, all relatives. On their land, the household cultivated cassava, maize, groundnuts and beans. Farm work was done by the household members, but labour was also mobilised through participating in male and female *gbisa.* The women prepared *ka* and cassava leaves for *gbisa* and it was not expected for them to provide fish or meat to the members of the work groups*.* The head was an active fisher and hunter. Around the homestead the household also grew a number of fruit trees. The household emphasised the value of a diverse diet and argued that they gave their children fish and fruit every day. They obtained safe drinking water from a water source prepared by CDI-Bwamanda.

#### Case 6

In this household, comprising 19 members, the head lived with his three wives. Other relatives in the household included three adult males, one adult female, six adolescents and five younger children. They had managed to clear several agricultural plots for cassava, groundnut and palm oil cultivation. In addition to household labour they relied on mobilising *gbisa* for land clearing and weeding. In the male work group fish and meat was served. The adult males participated in a “groundnut *gbisa*” and they had used the income from the *gbisa* to purchase a bicycle. They produced enough crops and cash to ensure that their members obtained a sufficient diet that usually included fish. In their compound they grew fruits and in between the meals children and adults ate bananas and pineapples. The household lived in the centre of the village and collected water from a borehole well drilled by CDI-Bwamanda.

#### Case 7

In this household there were 12 members where the livestock keeper lived together with his two wives, three adult males, two adult females, two adolescents and two younger children. The members of the household were all relatives. The household cultivated palm oil and coconuts in addition to the most common crops. It had also established a separate fruit orchard. In order to secure a regular supply of fish, one of the head’s wives specialised in fishing. The head participated in a *gbisa* that established a revolving fund providing cash on a consecutive basis to its members for capital accumulation and investment in livestock. To cover the *gbisa* investment, the household head used funds that his wives had saved from selling palm wine. With the capital received from the *gbisa*, the household invested in cattle. The *gbisa* group had later evolved into a group of livestock owners who cooperated on preventing livestock diseases. The adult members reported that their children were well-nourished because they could provide them with a diverse diet that included fish, meat and fruit. They also stated that good hygiene was important. In 2011 a project promoted good personal hygiene in the Bwamanda area and advised the household members to wash their hands before meals. They had attached a water bottle to a tree and water from this bottle was used for hand washing. Because the household had no access to safe drinking water, the head was in regular contact with the CDI-Bwamanda in the planning of drilling a new borehole well.

#### Case 8

This business household consisted of 13 members and the head lived with his three wives. Other relatives in the household included three adult males, one adult female, two adolescents and two younger children. They cultivated the most common staple crops while coconuts and oil palm were grown as cash crops. Besides employing household labour, they hired farm labourers and also engaged school students during harvest. Previously, CDI-Bwamanda had promoted cash cropping by purchasing crops from local farmers and shipping the produce to Kinshasa. At the beginning of the 1990s the household took advantage of this opportunity and with the profits made on cash crops they invested in a cigarette business. Income from the sale of cigarettes was invested in pigs, sheep and goats and, at a later stage, in cattle. The head believed that his children were healthy because, aside from *ka* and cassava leaves, they ate fish and a variety of fruit. The household had also followed the advice from the hygiene project and used water from a bottle attached to a tree for hand washing. It had access to drinking water from a borehole well drilled by CDI-Bwamanda.

### Social field analysis

We have identified four social fields that extend in social space each with their own characteristics. On the basis of our description of the Ngbaka socio-economic context it is possible to make linkages between activities and specific locations. Food production and consumption is associated with the household compound and household agricultural plots, access to external labour and capital with the neighbourhood, acquiring land with the village and social service provision with local NGO activities which again are linked to activities at the national and international level. Several characteristics are unique to these to these four fields. The household is the major unit for food production and consumption, division of labour is gender based and household composition influences its ability to produce sufficient and adequate food. Neighbourhood cooperation in the form of gbisa is characterised by being a reciprocal group for exchange of labour and provision of food and drinks to participants influence people’s willingness to participate in work groups. The gbisa plays and important role in capital accumulation. The village is associated with access to land and land is transferred from father to sons, living in a village and continuously cultivating the land is a precondition for access to land. The local NGO, CDI Bwamanda; in the absence of a strong state has become the main provider of social services. The NGO’s provision of social services establishes linkages between local activities and processes at higher levels. The identification of the fours social fields enabled us to conduct a cross-case analysis and compare households with malnourished children with those with well-nourished children.

#### The household

In our cases there are links between household size, composition and children’s nutritional status. Large households comprising many adults with relatively little pressure on productive members were able to broaden their economic activities and supply members with an adequacy of food, both in terms of quantity and variety. For example, household 7 included six adults and had managed to diversify its food production. The members specialised in growing fruit, making wine, herding cattle and fishing. In contrast, as indicated by case 1, nuclear families were particularly vulnerable and, when members became sick, the effect of ill health was food insecurity and malnutrition. It was not only size and dependency ratio that mattered, but also gender composition. The farmers practice shifting cultivation and clearing land relies heavily on male labour. As illustrated in case 3, shortage of male labour can result in failure to clear land, food insecurity and malnutrition. Among the Ngbaka it is women who are mainly responsible for weeding. Household 1 comprised only one woman and the poor harvest was primarily due to failing to properly weed the agricultural fields.

#### Inter-household cooperation – the Gbisa

Efficient food production does not only rely on household size and composition, but also on inter-household cooperation in the form of participation in *gbisa*. The cases show how households with well-nourished children managed to solve seasonal bottlenecks by mobilising agricultural labour through *gbisa* participation. In case 5, the household was in a positon to supply food desired by the group and by mobilising a work group it could solve the problem of shortage of male labour. In contrast, the household in case 3 was unable to provide the food needed to join a *gbisa*. The failure to take part in work groups was linked to an incapability to provide an adequate diet and malnutrition. *Gbisa* was also used for capital accumulation and revenues were used to strengthen household economic activities and thereby enhance food security. As illustrated in case 6, income from groundnut *gbisa* was spent on improving household transportation, while in case 7 profits were invested in cattle.

#### The village

In Bwamanda rights to land are closely linked to the village as a unit and access to land is maintained by staying in the village and continuously cultivating the land. In our cases, households with well-nourished children had access to labour and land, with wealthier households cultivating relatively large areas of land. In an area such as Bwamanda where there are few alternative income generating activities landlessness may result in food insecurity and child malnutrition. Household 3 illustrates this link between landlessness and malnutrition. The household which moved to the village of the malnourished child’s father failed to obtain agricultural land and had to rely on food as payment for work and a meagre income from selling palm oil.

#### The local NGO

Our study indicates that access to the limited services that exist is disproportionately associated with wealth. For example, in case 7, the household took advantage of efforts made by CDI-Bwamanda to promote the sale of maize. Profits made on cash cropping were used to expand economic activities with earnings being invested in petty-trade and livestock. The two better-off households (cases 7 and 8), also benefited from efforts to combat sleeping sickness, and as a result of the decline in this disease they could keep cattle. These two households also followed advice given by a hygiene project. Moreover households with well-nourished children gained from CDI-Bwamanda’s endeavours to improve access to safe drinking water while those with malnourished children had not. In case 4 the parents of the girl with kwashiorkor stated that malnutrition was a result of drinking contaminated water. Several factors constrain a household’s access to health services that could treat malnutrition. Local people have no means of transportation and parents must walk long distances to reach Bwamanda hospital. As indicated in case 2 it is difficult for poor households to pay fees for healthcare and the poor family had to sell their assets to cover hospital fees for treating the boy with marasmus. Bwamanda hospital also lacked food to properly treat malnutrition and the boy did not recover after he had been treated at the hospital.

## Discussion

In our study, access to vital resources for adequate food production was related to four social fields that generated conditions for social inequalities in nutrition. Households with sufficient land, enough labour and access to social services could ensure that their children stayed well-nourished. Households with well-nourished children also benefited from taking part in inter-household cooperation. In this study we identified four social fields that had consequences for food security and children’s nutritional status. First, household size and composition determined the household’s access to labour and hence ability to diversify food production. Second, through neighbour cooperation, in the form of *gbisa*, kin and neighbours could be mobilised for overcoming seasonal bottlenecks and for capital accumulation. Third, the village, which controlled access to land for food production and fourth, the local NGO providing different access to social services including agricultural support and health.

### The household

This study has shown how household organisation may relate to food and nutritional security. The Ngbaka live and work in an environment where resources are widely dispersed. In Bwamanda there are hardly any local means of transportation and farmers walk for several hours to reach their farms and fishing grounds. They also practice an intensive form of shifting cultivation. In accordance with the literature, our study demonstrates how in such environments larger households might be more efficient than small [58, 59]. Our findings also support the suggestion that in societies where the household is the production unit, households with a high pressure on the productive members are at risk of not being able to support themselves [60, 61]. Studies have investigated the relationship between family size and malnutrition and found that the odds for being malnourished are higher in large crowded families than in small families [62–65]. Whereas these studies relate family size to household crowding, our study has investigated how household size and composition influences productive activities. Our findings align with the notion that gender division of labour in agriculture has important implications for food production and nutrition [39, 66].

### Inter-household cooperation - the Gbisa

In accordance with reports from other areas our cases show that reciprocal work groups can play an important role in mobilising agricultural labour and solving seasonal bottlenecks [67–70]. Our findings show how the working groups could be mobilised in order to solve such tasks as land clearing and timely weeding. In order to mobilise reciprocal work groups, some reward is required - often food or alcoholic drinks [69]. This study shows how being unable to serve food required by the group members limit farmers ability to participate in g*bisa* and how this negatively affects access to labour, food production and hence food security. Reciprocal groups can also be organised for other purposes [71]. Among the Ngbaka such groups can play a role in capital accumulation and enhancement of food security.

Research has dealt with the relationship between access to social networks and children’s nutritional status and has found that participation, especially in large networks, is positively associated with child nutrition [72, 73]. Whereas these studies deal with how social networks can enhance mothers’ access to health advice, our study shows how networks in the form of inter-household cooperation may facilitate households’ access to agricultural labour and capital.

### The village

Our findings are in line with the literature that consider access to productive land to be one of the most important factors determining household food security and the landless to be vulnerable to food insecurity [74–76]. Land availability is considered to be a problem in the DRC and although there is a great potential to cultivate land in the DRC, farmers report difficulties in accessing land [36]. Quantitative studies have also found that access to agriculture land plays a role in determining children’s nutritional status and that children of agricultural workers are more likely to be malnourished than those of land owners [77, 78].

### The local NGO

As in many other areas in the DRC where public social services are minimal, an NGO delivers services in Bwamanda [37, 79, 80]. Our study indicates that the well-off had better access than the poor to the limited services that existed. Food insecurity and malnutrition is, as in other rural areas in the DRC, to a large extent related to distal factors including the government being unable to deliver basic services to rural areas such as agricultural support, infrastructure development, health, access to clean drinking water and education [35]. Other scholars have also demonstrated how macro-relations determine the development of severe child malnutrition. For example, an ethnographic account from rural Tanzania examined how fluctuations in the world economy, land shortage, population growth, social stratification and marginalisation were among the driving forces behind severe malnutrition [81].

### Social inequality in malnutrition

The literature has linked social determinants of malnutrition to income-related inequalities and documents pro-rich disparities in nutrition [12, 14, 82]. Household income and food prices are also closely related to food security. It has been shown that an inability to access food was largely determined by a low ability to purchase food rather than by local food production [83, 84]. However, since subsistence agriculture is the major livelihood in rural DRC, food security and inequalities in child nutrition is closely related to people’s capacity to produce enough nutritious food [36]. Research has identified maternal education, emphasising the importance of education higher than primary school, as one of the main factors that benefit child nutrition [14, 17, 18, 20]. Since many women in the study area were illiterate and few had education above primary level [85] we anticipate that maternal education had limited implications for inter-household differences in nutrition. Several studies from sub-Saharan Africa have investigated intra-household inequalities in the form of gender differences, but conclusions from these studies are contradictory [30–33]. Our field observations and discussions did not point towards any gender-based discrimination in food allocation and a study from Bwamanda did not find significant differences in nutritional status between girls and boys [51].

### Strengths and limitations

Studies on social inequality in malnutrition analyse Demographic and Health Survey (DHS) and Living Standards Measurement Study (LSM) data. Using data from a large number of low and middle-income countries research has been able to investigate the presence of and compared national and regional differences in socio-economic inequalities in malnutrition [15, 82, 86]. DHS and LSM apply a standard questionnaire approach on a set of predetermined variables and proxies for socio-economic status may not be representative for rural areas where people predominantly depend on agriculture [15, 87, 88]. This study has used different qualitative methods to gather open-ended information about a specific rural setting and our analysis has uncovered links between local social organisation and inequalities in nutrition. Our study uses few cases but the findings might be transferable to other population in a similar context in the DRC. The variables that we have identified may be applied in quantitative studies that can create quantitative evidence of the relation between the variables and nutritional outcomes in rural areas similar to Bwamanda. The combination of several methods including participant observation, semi-structured interviews and key informant interviews strengthens our study. By combining these methods we have managed to reveal how household organisation, inter-household cooperation, access to land, capital and social services relate to food security and nutrition. Data collection was carried out during three relatively short field work periods and continuing data collection with longer periods we could probably have gained new insights in social aspects of nutrition. We are well aware that our findings are based on a small sample and the results should be carefully interpreted when applied to other settings in the DRC. We therefore realise that social factors with implications for the development of kwashiorkor are somewhat ambiguous, and if we had included more kwashiorkor cases, the social etiology of this disease may have become clearer. The use of an interpreter and not transcribing the interviews also represent weaknesses of the study.

## Conclusions

Resources vital for food productions were associated with four social fields and access to these resources was unequally distributed creating social inequality in nutritional outcomes. Households could, by mobilising local institutions for inter-household cooperation, improve their food security. Children living in households where there was a great pressure on productive members were at risk of food insecurity and at danger of developing malnutrition. It is important that nutritional programmes involve institutions for inter-household cooperation to further improve food security and nutritional outcomes. These initiatives should address the problem of inequalities in service provision and making accessible social services that can improve food security and child nutrition in households with few resources in the form of labour, land and capital.
